# Epigenetic regulation and therapeutic targets in the tumor microenvironment

**DOI:** 10.1186/s43556-023-00126-2

**Published:** 2023-06-05

**Authors:** Zhuojun Xie, Zirui Zhou, Shuxian Yang, Shiwen Zhang, Bin Shao

**Affiliations:** 1 State Key Laboratory of Oral Diseases and National Clinical Research Center for Oral Diseases, West China Hospital of Stomatology, Sichuan University, No. 14, Section 3, South Renmin Road, Sichuan 610041 Chengdu, China; 2 Department of Oral Implantology, West China Hospital of Stomatology, Sichuan University, No. 14, Section 3, South Renmin Road, Sichuan 610041 Chengdu, China

**Keywords:** Tumour microenvironment, Epigenetics, Immune checkpoint inhibitors, Epigenetic drugs

## Abstract

The tumor microenvironment (TME) is crucial to neoplastic processes, fostering proliferation, angiogenesis and metastasis. Epigenetic regulations, primarily including DNA and RNA methylation, histone modification and non-coding RNA, have been generally recognized as an essential feature of tumor malignancy, exceedingly contributing to the dysregulation of the core gene expression in neoplastic cells, bringing about the evasion of immunosurveillance by influencing the immune cells in TME. Recently, compelling evidence have highlighted that clinical therapeutic approaches based on epigenetic machinery modulate carcinogenesis through targeting TME components, including normalizing cells’ phenotype, suppressing cells’ neovascularization and repressing the immunosuppressive components in TME. Therefore, TME components have been nominated as a promising target for epigenetic drugs in clinical cancer management. This review focuses on the mechanisms of epigenetic modifications occurring to the pivotal TME components including the stroma, immune and myeloid cells in various tumors reported in the last five years, concludes the tight correlation between TME reprogramming and tumor progression and immunosuppression, summarizes the current advances in cancer clinical treatments and potential therapeutic targets with reference to epigenetic drugs. Finally, we summarize some of the restrictions in the field of cancer research at the moment, further discuss several interesting epigenetic gene targets with potential strategies to boost antitumor immunity.

## Introduction

As one of the most threatening diseases hazardous to human health with limited therapeutic strategies, tumors are generally composed by a group of abnormally proliferative tumor cells which enmeshed in an extremely intricate ecosystem termed as the tumor microenvironment (TME). The TME is an aggregation of various non-neoplastic cells, extracellular matrix (ECM) that consists of multiple growth factors, chemokines and cytokines, along with the blood and lymphatic vascular networks [1]. Among which, the surrounding cells could be classified according to different phenotypes [2, 3], such as the immune cells including T and B lymphocytes and natural killer cells (NKs), the myeloid cells including dendritic cells (DCs), tumor-associated macrophages (TAMs) and myeloid-derived suppressor cells (MDSCs), the stroma cells including cancer-associated fibroblasts (CAFs) as well as some cancer stem cells (CSCs) and so on.

There is increasing evidence indicating that the favorable TME plays a crucial role in modulating the onset of carcinogenesis, tumor progression and metastasis [4]. As an example, it’s been demonstrated that TAMs could maintain an immune-suppressive microenvironment notably via the production of chemokines [5, 6], and the presence of CAF is a strong indicator of the poor clinical prognosis of various cancers [2, 7]. What’s more, the tumor cells could reversibly alter phenotypes of surrounding cells via cell-to-cell contacts, secretion of dissolvible cytokines and exosomes release, in ways to create the suppression of the immunosurveillance in TME [8, 9]. Consequently, developing therapeutic approaches targeting the TME has become one of the most attractive areas in cancer therapy [10].

Epigenetic regulation, identified as a heritable and reversible way to modify gene expression and chromatin structure without changing DNA sequence, is primarily initiated by the diverse profile of covalent modifications to nucleic acids and histone proteins [4, 11]. There are three main ways of epigenetic regulation: DNA methylation, histone modification, and non-coding RNA (ncRNA) alterations [12]. Epigenetic mechanism is widely acknowledged to affect all aspect of tumor progression [11, 12], further regarded as an essential hallmark of malignancy by breaking the balance between multiple oncogenic and tumor suppression gene pathways in cancer cells [13–15]. Furthermore, epigenetic modification also takes place in TME, contributing to the evasion of immunosurveillance by influencing the differentiation, infiltration, and activation of immune cells [16], such as switching immunogenic DCs into immunoregulatory DCs (regDCs), and promoting the polarization of TAMs from M1 to M2 phenotype [8]. Accordingly, epigenetic regulation, as a key modulator in the process of cancers, has been nominated as a potential clinic therapeutic approach in cancers.

In this review, we focus on the latest epigenetic events happened to TME, summarize the clinical treatments and potential therapeutic targets towards various cancers based on the epigenetic machinery. We outlook some prospective epigenetic gene targets, also discuss some of the limitations and doubts in the field of cancer research and epi-regulatory studies currently.

## Fundamental modes of epigenetics

**Fig. 1:**
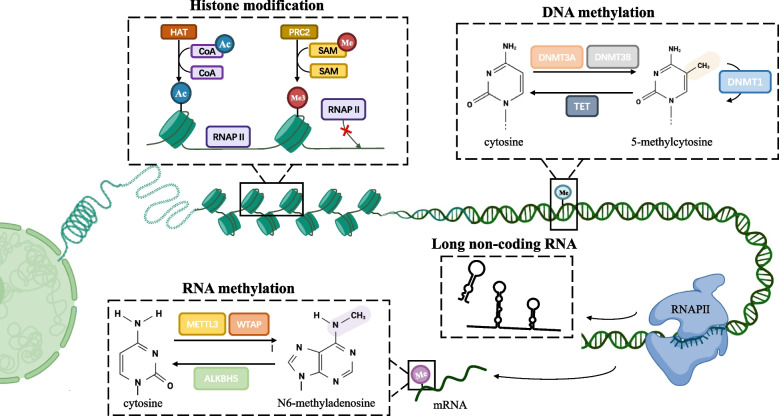
Basic regulatory mechanisms of epigenetic modification. Histone modification, including acetylation and methylation, occurs on the N-terminal tails of H3 and H4 subunits and leads to chromatin configuration alteration and subsequent transcriptional blockade. DNA methylation is a highly dynamic process in which DNMTs transfer methyl groups onto the 5’ position of cytosine in the CpG islands, whereas enzymes in the TET family can remove it. RNA methylation often occurs on the 6’ position of adenosine, and is also reversible under the modulation of the writer complex (METTL3, METTL14 and WTAP) and the erasers (FTO and ALKBH5) Non-coding, including miRNAs, lncRNAs and so on, can interfere the protein translation and participate in the pro-translational modification of proteins

Due to the dramatically developed global proteomic and genomic technologies over the past few years, epigenetics has grown into one of the most active research projects in biology, involving numerous physiological and pathological processes. Epigenetic regulations have been thoroughly studied and summarized in various reviews [4, 13, 16–19], mainly consisting of the followings (Fig. 1). We’re going to briefly summarize some fundamental modes of epigenetics, especially concentrating on some classical regulatory molecules and pathways.

### DNA and RNA methylation

DNA methylation commonly happens with the covalent addition of a methyl group and is mostly transferred from S-adenosylmethionine (SAM) onto the C5 position of the cytosine ring (5mC) of DNA [20, 21]. In the mammalian genome, this modification is generally enriched within cytosine- guanine dinucleotide (CpG) site, which is present throughout the genome but majorly situated in gene regulatory regions [22, 23] such as gene promoters or transcription start sites [24]. The methylation of CpG islands, consisted by CpG sites and abundant repetitive sequences, is closely associated with the deregulation of cellular pathways and gene expression, particularly gene silencing, in many biological processes and various diseases including cancers [17]. For example, the hypermethylation of CpG islands in the promoter regions of tumor suppressor genes (TSGs) leads to TSG silencing, eventually result in tumorigenesis [25].

The state of DNA hypermethylation is catalyzed by DNA methyltransferases (DNMTs), including DNMT1, DNMT3A/3B along with DNMT3L. Among them, maintaining methylation on the new DNA strand predominantly during DNA-replication S phase is medicated by DNMT1, while de novo DNA methylation in the unmethylated genomic regions principally during embryonic development is catalyzed by DNMT3A/3B with the aid of DNMT3L [26, 27], the latter one who could expand the methylated pattern on DNA sequences and simultaneously activate the enzymatic ability of DNMT3A/3B [28]. On the contrary, the removal of the methyl group is catalyzed by the ten-eleven translocation (TET) family triggered by either active or passive mechanisms, known as the synergistic activation with the thymine DNA glycosylase (TDG) and the base-excision repair (BER) machinery [29, 30] or the DNA replication-dependent passive pathway [31], respectively. Therefore, as one of the most common epigenetic regulations, DNA methylation on both global and specific sites has been broadly considered as the promising biomarkers to forecast tumor phenotypes or overall survival or patient prognosis [32–38].

Except for DNA methylation, RNA methylation has gained increasing attention. Although all nucleotides composed RNA could be modified and interact with each other [39], the most abundant target is *N*6-methyladenosine(m6A), which refers to the methylation at the sixth nitrogen atom of RNA base A. Generally speaking, the m6A modification is enriched near stop codons of transcripts and in conserved regions of the 3′-untranslated regions (3′-UTRs) [40]. This process is catalyzed by m6A-methyltransferases, such as the complex of Methyltransferase-like (METTL)-3/14, Wilms’ tumor 1-associated protein (WTAP) and so on [41, 42], while de-catalyzed by the RNA demethylase α-ketoglutarate-dependent dioxygenase homolog 5 (ALKBH5) [43]. Except for those “writers” and “erasers”, the m6A group should be identified by various “readers” to exert multiple functions, which is mainly undertaken by YT521-B homology (YTH) domain family members including YTHDF1, YTHDF2, YTHDF3, YTHDC1 and so on [44]. The presence of m6A exerts a wide range of effects on RNA stability and function, subsequently modifying cellular processes and altering pathological conditions including the development of cancer [45, 46].

### Histone modifications

As the fundamental subunits of chromatin, nucleosome encompasses four subtypes of histone proteins including H2A, H2B, H3 and H4 [47]. The posttranslational regulations of histone, containing acetylation, methylation, phosphorylation, ubiquitination and so on [48], mostly happened to histone H3 followed by H4 [49], could alter the accessibility of genes for the transcriptional machinery and ultimately modulate gene expression by disrupt chromatin structures [50]. Among those histone modifications, histone acetylation and methylation is the most pivotal and common types.

Catalyzed by histone acetyltransferases (HATs), the acetylation of histone lysine residues undergoes the convertible addition of acetyl groups in the lysine-rich N-terminal tails, consequently loosens the DNA-histone bonds and promotes the binding of transcription factor, mostly associated with the activation of transcriptional activities [13, 51]. On the contrary, catalyzed by histone deacetylases (HDACs), the deacetylation of histone facilitates compact wrapping of the DNA duplex around histones, subsequently linked to gene repression [13] and closely correlated with poor clinic prognosis, which has been widely verified in various cancers [52, 53]. According to the homology of sequence and the mechanism of catalyze, so far 18 mammalian HDACs have been identified and preliminarily classified into four classes: class I (HDAC1-3 and 8), class II (HDAC4-7, 9 and 10), class III (Sirt1-7) and class IV (HDAC11) [54].

Except for acetylation, histone methylation, the third major type of epigenetic modification [55], is mediated adversely by two major classes of enzymes respectively called histone methyltransferases (HMTs), which adding the methyl group to specific histone lysine or arginine residues, and histone lysine methyltransferases (HKMTs), which is opposed to this process [56]. Multiple methylation status and dynamic histone loci could elicit different outcomes, either repress or active gene expressions [57], and the dysregulation of which might play the fundamental roles in abnormal cellular proliferation, invasion, and metastasis during disease progression [58–60]. As an example, it’s been reported that the downregulation of lysine methyltransferase EZH2 resulted in the reduction of histone H3 lysine 27 (H3K27me3) in lung cancer [61], while the activation of histone H3 lysine 4 (H3K4) and the subsequent elevation of the antioxidant signaling pathway in prostate cancer was discovered owing to the lysine methyltransferase 7 (SetD7) [62].

### Non-coding RNAs

Constituted by long non-coding RNAs (lncRNAs), microRNAs (miRNAs) and circular RNAs (circRNAs) [63], ncRNAs are indispensable epigenetic regulators for various physiological and pathological activities via impeding the transcription of messager RNAs (mRNAs) or binding to proteins [64]. As the most thoroughly studied ncRNAs, miRNAs modulate gene expression through repressing translation or promoting degradation of the target mRNA by binding to 3′-UTR of which, thus have been considered as the critical cogs in the onsets and maintenance of cancers [65–67] and correlated with poor clinical prognosis [68, 69]. It’s been well demonstrated that miRNAs have engaged in remodeling TME by being encapsulated within extracellular vesicles (EVs) or exosomes [70]. For example, EVs released from CAFs could promote the migration and the invasion of oral squamous cell carcinoma (OSCC) [70, 71]. As a consequence, it’s worth noting that tremendous tumor treatments involving nanoparticle-conjugated miRNA mimics have made significant progress [72], which would be detailly discussed in the following.

Except for miRNA, compelling evidence have displayed that lncRNA have close correlation with epigenetics by taking advantage of chromatin modification or other types of ncRNA, inducing aberrant gene expression and implicating tumorigenesis [73]. Furthermore, it’s been demonstrated that various lncRNAs have ability to regulate chemotherapy via modulating pharmacological process of chemical drugs in several digestive system cancers [74–76]. Wu., et al. [77] has verified that bladder cancer associated transcript-1 (BLACAT1), a novel gene regulator lncRNA, could provoke the oxaliplatin(OXA)-resistance in gastric cancer through combining with miR-361, providing a novel insight and a potential therapeutic mechanism towards cancer chemoresistance.

**Fig. 2:**
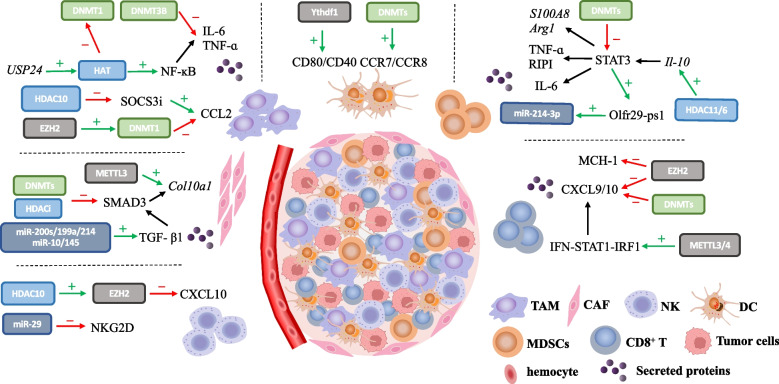
Epigenetic regulation of cell populations in TME. In TAMs, DNMT3A and DNMT3B suppress the secretion of IL-6 and TNF-a whereas HATs can rescue it by activating NF-κB pathway. Similarly, HDAC10 and EZH2 negatively regulate the expression of CCL2 by inhibiting SOCS3i and activating DNMT1, respectively. TGF-β1 signaling, the major force that drives CAF activation, is mediated by DNMTs, HDACs and several miRNAs. Of note, the m6A writer METTL3 can increase Col10A expression and promotes acquirement of myofibroblast characteristics. The cytotoxicity of NK cells is impaired due to EZH2-mediated CXCL10 decrease and miR-29 induced NKG2D deficiency, whereas DCs are activated by the m6A reader YTHDF1 and DNMTs. MDSC expansion and function rely on STAT3 activation, which can be suppressed by DNMTs or promoted by HDAC6/11. EZH2 and DNMTs inhibit MHC-I and CXCL9/10 on CD8^+^ T cells whereas METTL3/14 can activate IFN-γ signaling

Collectively, diverse epigenetic mechanisms have been considered as a decisive factor in the tumorigenic processes, contributing to tumor initiation and propagation, immune escape, metastatic, angiogenesis and prognosis [78–80]. To date, it’s emerging that both individual and combinatorial use of epigenetic drugs have shown prominent clinic therapeutic effect, while it’s believing that a plethora of novel epigenetic strategies could pose a promising future to oncotherapy [81]. The detailed epigenetic modifications and epigenetic drugs targeted multiple components in TME within various tumor types would be discussed and summarized in the following (Fig. 2).

## Epigenetic regulations in TME

### Epigenetic modulation in tumor cells

Epigenetic phenomena occurring directly in various tumor cells have attracted great attention all along, which could straightly dominate comprehensive aspects of tumor biology via impacting the phenotypes of tumor cells or the interaction with other components in TME. However, due to the multiple epi-regulated modes in various cancer types, it’s going to briefly illustrate some typical examples here (Table 1).

**Table 1:** Epigenetic modulation in tumor cells

Epigenetic regulations	Epigenetic targets in various cancer types	Contributed to tumor biology	References
DNA methylation	• Global hypermethylated DNA in lung, renal, colorectal, pancreatic cancers and melanoma • Hypermethylated: CD247, LCK, and PSTPIP1 in lung cancer; nine genes including LINC01555, GSTM1 in colorectal cancer; Foxp3 in hepatocellular cancer; SOX family in breast cancer; JAK3 in prostate cancer and melanoma; HLA-1 in lung cancer	biomarkers predicting tumor phenotype or immune or prognosis	[37, 82–89]
	• Hypermethylated: FAM107A in pan-cancer analysis; IL-1 axis in pancreatic cancer; IL-15 axis in gastric cancer; JAK3 in prostate cancer and melanoma; HLA-1 in lung cancer	directly lead to tumorigenesis	[34, 88–91]
	• Hypermethylated: immunosuppressive molecules such as PD-L1 in pan-cancer analysis; SOX18 in breast cancer; IL-15 axis in gastric cancer; Foxp3 in hepatocellular cancer; JAK3 in prostate cancer and melanoma • Hypomethylated: KIF2C in oral cancer; MGST1 in hepatocellular and gastric cancers; TIGIT in nasopharyngeal and uterine cancers	related to the remodeling of TMA, including the immune cells infiltration	[34–36, 86–88, 92, 93]
RNA methylation^a^	• Global hypermethylated DNA due to upregulated m6A writers and readers in renal cancer • Hypomethylated WTAP in gastric cancer	correlated with tumor grades and prognosis	[94–96]
	• Global hypermethylated RNA due to the chronic exposure to Cr(VI) and upregulated METTL3 in lung cancer • Hypermethylated BCL-2 catalyzed by METTL3	ontogenetic functions related to tumor biology	[97, 98]
	• Global hypermethylated RNA in renal cancer • Hypomethylated: WTAP in gastric cancer; PD-L1 in hepatocellular cancer; ALKBH5 in cholangiocarcinoma and melanoma	related to TME diversity and complexity, including immune cell infiltration	[94–96, 99–101]
Histone modification	• Acetylated: SMAD3 in breast cancer; upregulated potential-histone-acetyltransferase CLOCK in glioblastoma • Deacetylated: p53 due to the interaction with HADC1/8 in T-cell lymphomas; loss of H3K27ac at master transcription factors (TFs) of epithelial phenotype in glioblastoma	oncogenic function, decide the malignant phenotype	[102–106]
	• Acetylated: H3 promoter at PD-L1 in breast cancer; upregulated potential-histone-acetyltransferase CLOCK in glioblastoma • Deacetylated: loss of H3K27ac at master transcription factors (TFs) of epithelial phenotype in glioblastoma; high expression of HMGB3 due to reduced HDAC3 in breast cancer • Hypermethylated: H3K27me3 at MHC-I in drug-resistant vitro models • Hypomethylated: loss of H3K9me2 due to upregulated histone demethylase KDM3A in pancreatic; loss of H3K9me2 and H3K4me2 due to upregulated histone demethylase KDM1A in pan-cancer analysis	related to the immunosuppression, influence the efficacy of immunotherapy	[104–111]
ncRNA	• Downregulated miR-325 in T cell leukemia • Dysregulated lncRNAs (LINC00393, KB-1836B5.1, RP1-140K8.5, AC005162.1, and AC020916.2) in breast cancer subtypes	related to tumorigenesis	[112–115]
	• Upregulated: miR-93/106b suppress PD-L1 and CXCL12 in pan-cancer analysis; lncRNA u50535 promote CCL20 in colorectal cancer; lnC00301 targeting TGF-β in lung cancer • Downregulated miR-211 due to m6A modification catalyzed by METTL14 in T cell leukemia	related to the immunosuppression, influence the prognosis	[116–118]

^a^ Various research on RNA methylation in tumor before 2018 have been summarized in [119]

The influences that DNA methylation occur in cancer cells on tumor progression are mainly divided into two modes. Firstly, it’s been widely proven that aberrant methylation happened in specific gene sites may directly promote carcinogenesis via diverse mechanisms, such as in acute myeloid leukemia (AML) [120] and hepatocellular carcinoma (HCC) [121]. Additionally, TME homeostasis is also significantly impacted by abnormal DNA methylation in cancer cells, resulting in disordered interactions among various extracellular components [101, 122].

Except for DNA methylation, RNA methylation also plays an indispensable role in cancer biology, which has been detailly concluded and summarized in a 2018 review [123]. However, thanks to the research boom in recent years, multiple newly-discovered RNA methylation mechanisms have been clearly reflected, especially m6A modification. As a typical example, Fang, J., et al. has proven that almost all m6A methylation regulators were greatly correlated with the grades and stages of kidney renal clear cell carcinoma [90], and numerous research have revealed the nonnegligible role of m6A played in the progression of tumorigenesis via multiple mechanisms [102, 124, 125].

As for histone modification, the carcinogenicity mechanisms of both histone acetylation and methylation existing in cancer cells are similar to DNA epigenetics, which could be classified as the direct impact on oncogenic regulations, or the profound influence on surrounding components, especially the immune cells such as the CD8^+^ T cell in human ovarian cancers [126]. In addition to the classic epi-regulatory enzymes, more and more modulars in cancer cells have been discovered to show the potential epigenetic regulatory activities. In glioblastoma, for an instance, a gain-of-function screen revealed that the circadian locomotor output cycles kaput (CLOCK), generally enhancing the self-renew activities in the normal cells, exhibited as the potential histone acetyltransferase, subsequently regulating glioma cell proliferation and migration and supporting an immune suppressive TME [127].

Although studies targeting ncRNAs modification towards tumor cells needed further exploration, diverse research have illustrated that ncRNAs, notably miRNAs, appear to have a critical function in cancer cells. According to the landscape of epigenetically dysregulated ncRNAs in three breast cancer subtypes, ncRNAs contributed to biological functions that represent as a hallmark of different subgroups [103], hoping to become the potential biomarkers. Furthermore, upon the thorough insight into pan-cancer models, Cioffi, M., et al. found that miR-93 and miR-106b diminished tumor immunity and metastasis of cancer cells respectively by targeting the programmed death ligand 1 (PD-L1) which is known as the immune checkpoint inhibitors that could inhibit immune response [128], and downregulating the production of the chemoattractant CXCL12 [129], uncovering the strong immunosuppressive and metastatic phenotypes built by ncRNA.

### Epigenetic modulation in cancer-associated fibroblasts (CAFs)

Fibroblasts, generally quiescent in normal tissues, could be activated and subsequently turned into myofibroblasts in tissue repair and scar formation [130], therefore playing a prominent role in the progression of various tumors which are classically considered as “wounds that do not heal” [131], becoming the most abundant component in TME and having an essential influence on tumor initiation, angiogenesis, metastasis and anticancer therapy [132].

The functional diversity and phenotypic heterogeneity of CAF has been extended with the help of epigenetics study, and the most in-depth investigation is the conversion of quiescent fibroblasts into activated CAF in various cancers [97, 133–135], while one of the most classic down-streaming targets is TGF-β1 signaling and its critical mediator SMAD3. With the recognition that various genes responsible for collagen synthesis and fibroblastic phenotypic transformation are activated by TGF-β1 in Smad3-dependent manner [136], it’s been uncovered that the global hypermethylation especially promoter hypermethylation-associated SMAD3 silencing could be attributed to the neoplastic transformation in lung cancer [104], and DNMT3B-catalyzed hypermethylation at miR-200 s promoter could establish CAF activation by sustaining autocrine TGF-β1 in breast cancer [137]. Furthermore, various miRNA in CAF, such as miR-199a/214 in the pancreatic stellate cells (PSCs) [138], miR-101 in hepatocellular carcinoma [139] and miR-145 in oral cancer [140] could modify TGF-β1 signaling and mediate CAF, contributing to the tumor angiogenesis and migration. Additionally, it’s been newly demonstrated that the expression of COL10A1, one of the abundant collagen families controlled by SMAD3, was increased by m6A modification via elevated METTL3 in CAF, thus accelerating cell proliferation and repressing apoptosis of the lung cancer model both in vitro and in vivo [141]. Moreover, applying an HDACs inhibitor CUDC-907 to lung tumor could notably repress the phosphorylation level of SMAD2/3 and promote histones acetylation, ultimately blocking lung fibrosis [142]. In conclusion, TGF-β1/SMAD3 signaling has become a potential therapeutic target in diverse cancers that should be tested in future studies.

Except for miRNAs mentioned above, there is multiple evidence proving that CAF share complex crosstalk with cancer cells through various miRNAs [143], among which the most abundant and well-studied miR-21 and miR-200 will be summarized here. The miR-21 is regarded as a pivotal prognostic marker in lung adenocarcinoma, whose expression in CAF is inversely correlated with patient survival [144]. The overexpression of miR-21 could activate CAF by driving its trans-differentiation [9] as well as the metabolic alteration [145]. Despite the direct effect on CAF, it’s been extensively studied that miR-21 modulates tumor biology including metastasis and invasion [146], such as the upliftingly proliferative capacity of the pancreatic ductal adenocarcinoma (PDAC) cells caused by upregulating expression of various modulars secreted from CAF which highly expressed miR-21 level, such as matrix metalloproteinases (MMP)-3/9, Platelet-derived growth factor (PDGF), and chemokine (C–C motif) ligand 7 (CCL-7) [147]. Meanwhile, the miR-200 family members also exhibit key roles in tumorigenesis and correlate tightly with disease prognosis, such as the accumulation of CXCL12 was miR-141/200a-dependent and related to the immunosuppressive functions of CAF in high-grade serous ovarian cancers (HGSOC) [148].

### Epigenetic modulation in myeloid-derived suppressor cells (MDSCs)

The pathological process of tumors involves a myriad of complicated interactions between cancer and immune cells, where immune cells are responsible for both pro-tumorigenic and anti-tumorigenic regulation [149]. MDSCs, a heterogeneous cluster of immature myeloid cells, mainly exhibit the immunosuppressive role by suppressing the proliferation and anti-tumor function of T cells or NK cells to promote tumor development [150]. Thus, the deep investigation into the activation, recruitment and expansion of MDSCs via the epigenetic regulation might be the key to find out the immunological mechanisms in TME, helping to develop immunotherapies with improved therapeutic outcomes.

It's been discovered that MDSCs present a global DNA hypomethylation profile in ovarian cancer [151], and the repression of genes related to an immunogenic phenotype during MDSCs generation implies the establishment of a function-specific DNA methylation pattern. Among the complex DNA methylated targets, an elevated expression of transcription (STAT) 3, known as a master transcription factor mediating a wide range of cell functions of MDSCs [24], has been widely defined as the result of hypomethylated promoter silencing due to the attenuated DNMT3A/3B in various cancers including lung, pancreas and renal cancer [152–155]. Intriguingly, it’s been verified that after treating MDSCs with interleukin (IL)-6 in the vitro tumor-bearing mice model, the activation of STAT3 further elevated expression of DNMT1 and DNMT3B, resulting in the decreased downstream signaling pathway such as tumor necrosis factor-α (TNF-α)-induced and receptor interacting serine/threonine kinase 1 (RIP1)-dependent necroptosis, eventually promoting MDSCs survival and accumulation [99].

In addition to DNA methylation, IL-6, a potent inducer in tumor aggression and MDSCs expansion [156], could alter RNA methylation in MDSCs to mediate its function. Shang, W., et al. revealed that IL-6-mediated m6A upregulated a lncRNA pseudogene, Olfr29-ps1, which corrected negatively with miR-214-3p, thus modulated immunosuppressive function and differentiation of MDSCs in the inflammatory tumor environment [157]. What’s more, the tight correction between IL-6 signaling pathways and epigenetic regulations has furthermore disclosed by a clinical investigation of colorectal cancer, displaying that in tumor tissues refraining HDAC activation or neutralizing IL-6 substantially inhibits the expression of genes related to immunosuppression and chemotaxis of MDSCs [158]. Same as IL-6, IL-10 is considered as a key modulator of immune responses in MDSCs, whose expression is notably dependent on histone modification. It’s been widely verified that HDAC11 and HDAC6 were cooperatively recruited to the same region of the IL-10 gene promoter in antigen-presenting cells(APCs), as HDAC11 had been identified as a negative modifier of MDSCs expansion and function in mice model while HDAC6 had a contrary effect [159], thus controlling IL-10 transcription and the subsequent STAT3 expression, which targeted to amplify the immunosuppressive molecular S100A8 and Arginase 1 (Arg1) in MDSCs [160]. Thus, for efficacious immunotherapies, a better and deeper understanding of the relationship between those cytokines and signaling pathways regulated by epigenetics is still required.

Despite the direct-regulator miRNAs in MDSCs previously mentioned, multiple ncRNAs intricately modulate MDSCs through various pathways [161]. Here, we display some recent research. Firstly, the functions of MDSCs could be altered via exosomes containing miRNAs secreted from the cancer cells, such as glioma-derived exosomes can enhance the differentiation and propagation of MDSCs by miR-29a, miR-92a, miR-10a and miR-21 targeting to various proteins [162, 163], while the conversion and immunosuppressive phenotypes of MDSCs could be promoted by melanoma extracellular vesicles (EVs) including a group of miRs (miR-146a, miR-155, miR-125b and so on) [164]. Secondly, as Labib Salem, M., et al. [112] has addressed that by detecting the total RNAs in acute lymphoblastic leukemia (ALL) patients, many miRNAs were shared between MDSCs and regulatory T cells (Treg) and most of them were closely related to the immunoregulatory pathways, which reveals the role of miRNA as the biomarkers for the immunosuppressive TME. For example, the suppression of MDSCs was associated with the restoration of miR-195 and miR-16 by blocking PD-L1 in TME, while the elevated levels of those miRNAs were positively associated with the recurrence-free survival of prostate cancer patients [165].

### Epigenetic modulation in Tumor-associated macrophages (TAMs)

Among the most abundant immune cells in TME, macrophages are originally antitumoral owing to the phagocytosis and direct tumor-kill [166], but once a tumor has progressed past an initial stage, they switching to play an exactly contrary role commonly deemed TAMs [167]. Depending on converging signals from the cellular environment, macrophages obtain a serious of activation phenotypes, broadly described as M1 macrophages, normally considered as antitumor, and M2 macrophages who contribute to pro-tumorigenesis via inducing immune evasion as well as supporting tumor cell invasion and expansion [168, 169]. As a result, the recruitment and polarization of TAMs, mediated by epigenetic regulation in both TME and malignant cells, is crucial for further exploration for the immunological therapy and antitumor outcomes.

Recent studies on epigenetic modification relevant to TAMs mainly focus on its regulation of phenotypic transformation and polarization. Inhabitation of DNMT3B has been recognized to play an essential role by repressing Arg1, one of M2 macrophage markers further related to its immunosuppressive functions [170], as well as blocking some inflammatory molecules such as TNF-α and IL-1β [171]. These actions significantly decrease immunosuppressive M2 macrophages populations while increase tumor-killing M1 macrophages and NK cells [172]. What’s more, with respect to RNA methylation, numerous vitro models and mice models have confirmed that by reducing the infiltration of M1 macrophages and Treg cells in TME, Mettl3-deficient mice displayed faster tumor growth and attenuated therapeutic efficacy of PD-1 checkpoint blockade. Following m6A sequencing revealed that YTHDF1-mediated translation of Sprouty-related, EVH1 domain containing 2 (SPRED2), which is responsible for inducing NF-κB and STAT3 through the ERK pathway, was impaired by the loss of METTL3 [173, 174], highlighting the m6A machinery as a potential cancer immunotherapy target.

M2 macrophages abundance in TME is also regulated by histone modification. As an example, USP24, a deubiquitinating enzyme upregulated in M2 macrophages of lung cancer cells, could stabilize HAT p300 thus increase H3 acetylation and NF-κB while decrease DNMT1, subsequently elevating TNF-α and IL-6 transcription, ultimately resulting in cancer malignancy [175]. Furthermore, CCL2, along with its ligand CCR2 expressed by monocytes/macrophages and T cells, exhibits underlying effect on TAM survival and function polarization, particularly responsible for immunosuppression [176]. In pancreatic ductal adenocarcinoma, HDAC5 could repress the suppressor of cytokine signaling 3 (SOCS3), a negative regulator of chemokine CCL2, contributing to elevated CCL2 and following recruitment of M2 macrophages [177]. In lung cancer, CCL2 expression was attenuated by EZH2-mediated H3K27me3 in the enhancer regions and DNMT1-mediated DNA methylation in the promoter regions [178], impeding M2-like phenotype of macrophages thereby facilitating metastasis and infiltration [179]. The same impact of CCL2/CCR2 axis on TAM further on tumor migration and invasion has also been demonstrated in breast cancer [180], constructing as a potential antitumor therapeutic target.

Regarding miRNAs, the regulatory mechanism towards TAM could be broadly divided into two categories. Firstly, it’s been widely reported that macrophages take up the exosomes or EVs contained miRNA which are secreted from malignant cells to alter its infiltration and polarization in TME [175, 181–184]. For example, excreted from breast cancer, miR-183-5p enhanced NF-κB signaling and promoted IL-1β, IL-6, and TNF-α expression in TAM [184]. Besides, macrophages could release the exosomes or EVs to remodel TME and modulate tumor progression [185–191], as miR-95 in TAM-derived exosomes could be directly uptaken by prostate cancer (PCa) cells and subsequent bound to JunB, a transcription factor governed cell development, consequently improving the proliferation, invasion and epithelial-mesenchymal transition of cancer cells, related to worse clinicopathological feature [192]. To draw a conclusion, the communication within cancer cells, TAM and other components in TME plays a critical role in tumor development and miRNAs occupy an essential position in this process.

### Epigenetic modulation in dendritic cells (DCs)

Being one of the most critical members of antigen presenting cells (APCs) for adaptive immune response, DCs are responsible for the activation and maintenance of tumor cytotoxicity by T cells [193]. Although the function and phenotype of activated DCs is generally determined by co-stimulatory molecules (CD80, CD86) and chemokine receptors such as CCR7 [194, 195], the immunological function of DCs could be influenced by multiple factors within TME, which is partly modulated by epigenetic regulations and being considered as the potential targets to elevate immune surveillance and enhance anti-tumor immunity [196].

As 5-AZA, a classical DNMT inhibitor, conjunct with mice vaccination or DCs fusions could similarly elicit anti-tumor immunity via elevating the prefoliation of CD8^+^ T cells and increasing overall survival in diverse tumor models [197, 198], the strong connection between DNA methylation and immune-related genes in DCs has been uncovered [199]. Additionally, m6A modification in CD80 and CD40 in DCs could be recognized by Ythdf1, the m6A readers, therefore enhancing the translation of those essential immune transcripts that promotes DC activation and DC-induced T cell response in the physiological condition [200, 201]. In vitro model, CCR7 stimulation could promote a lncRNA named lnc-Dpf3 by removing m6A modification, therefore decreasing CCR7-mediated DCs migration along with attenuated adaptive immune responses [202, 203]. Moreover, genome-wide analyses of melanoma models revealed that H3K4me3 was increased on DC maturation genes such as CCR7 and CD86 [204], while De Beck, L., et al. [205] fund that a dual inhibitor of histone methyltransferase G9a and DNMTs could jointly increase the therapeutic efficacy of DC vaccination by arresting tumor cell cycle and delaying tumor growth of tumor-bearing mice, indicating the critical role of the cellular epigenetic regulation in modulating DC phenotypes, maturation and antitumor responses.

Same to the regulatory mechanism of miRNAs towards TAM, DCs also take up the exosomes or EVs secreted from malignant cells. It’s reported that after treating DCs with the pancreatic cancer-derived exosomes, ENST00000560647 was differentially expressed, which predicted to interact with miR-323-3p to directly suppress the invasion and metastasis of cancer cells [206]. Further investigation is required to explore the epigenetic regulatory function of ncRNAs in DCs.

### Epigenetic modulation in tumor-infiltrating lymphocytes (TILs)

Regarding as one of the most essential components of the adaptive immune system, TILs are surrounding the cancer cells and become the foundation of current immunotherapy and the determination of the antitumor outcomes [207]. Within the complicated phenotypes and functional properties of TILs, NK cells and T cells, including CD8^+^ T cells, CD4^+^ T cells and Treg cells, are most common and closely correlated with the oncological prognosis [208]. Among them, NK cells could rapidly mediate cytotoxicity and involve in various tumor progression, metastasis and survival [209, 210]. CD8^+^ T cells are capable of producing antitumor cytokines and cytotoxic ligands including interferon-γ (IFN-γ) and TNF-α maintain their antiviral and antitumor functions [211], while Treg cells, whose high content is usually correlated with worse survival in multiple solid tumors, are renowned for their immunosuppressive role by modifying APCs and producing suppressive cytokines including IL-10, IL-35, and TGF-β to counteract CD8^+^ T cells [212]. Therefore, the in-depth investigation into the epigenetic regulatory mechanisms of TILs is critical for establishing effective antitumor immunotherapy.

As TME could be qualitatively characterized by gene expression-based methods, methylation data are now emergingly applied for molecular diagnosis, for example, the estimation of TILs abundance, infiltration and anti-tumor immune response have been successfully established by analyzing the global differential methylation in the central nervous system (CNS) tumors and glioblastomas [83, 95, 213]. Despite the global DNA methylation, gene expression of Th1 chemokines CXCL9 and CXCL10 was mediated by EZH2 and DNMT1, dampening CD8^+^ T cell infiltration in ovarian cancers [126], and the demethylation state of promoters in Th1 was proven as a significant mediator to IFN-γ production in colon cancer lymphocytes [214]. In addition to T cells, it’s been verified that hypermethylated CpG at the promoter locus induced the less mature subtype of tyrosine kinase 2 (TYK2), modifying the maturation and antitumor activity of NK cells in vitro model [198]. Those studies jointly indicate that DNA methylation role as a potential biomarker to significantly impact the infiltration and function of immune cells.

As a pro-inflammatory cytokine abundantly produced by TILs and in turn mediates their functions, IFN-γ is also controlled by RNA methylation. Inhibition of m6A in colorectal cancer via METTL3/4 deficiency resulted in the upregulation of IFN-γ-STAT1-IRF1 signaling through stabilizing the STAT1 and IRF1 mRNA, increasing secretion of IFN-γ, CXCL9, and CXCL10 further promoting infiltrating CD8^+^ T cells, contributing to the enhanced response to anti-PD-1 treatment [119]. Meanwhile, decreased m6A modification in Treg cells blocked SOCS mRNA, inducing the suppression of IL-2-STAT5 signaling pathway and ultimately losing the suppressive function of Treg cells over T cell proliferation which is beneficial to cancer immunotherapy [117]. What’s more, it’s been illustrated that RNA methylation also gives rise to phenotypic stability [89] or glycolytic-epigenetic reprogramming [96] in TILs.

With reference to histone methylation, EZH2 along with its target, H3k29me3, is playing an essential role in modulating the immunological functions of TCLs. The recruitment of T cells in human colon cancer tissue was impacted by CXCL9 and CXCL10, whose expression is regulated by H3K27me3 repression marks [126, 215]. In consistent to those studies, disrupting EZH2 activity in Tregs could selectively deplete T cells and alter their functions in murine colorectal tumor, enhancing the recruitment of CD8^+^ and CD4^+^ T cells and remodeling the TME [118], whilst high expression of EZH2 was shown to improve the escape from immunotherapy in neuroblastoma through transcriptional downregulation of major histocompatibility complex (MHC)-1 genes by H3K27me3 modification [216]. Besides, the cellular cytotoxicity and immunoregulatory function of NK cells are profoundly impacted by EZH2/CXCL10 axis as well [217–219]. As an example, Bugide, S., et al. [220] demonstrated that in hepatic tumor cells, HDAC10 was sufficient for EZH2 recruitment to the CXCL10 promoter, resulting in CXCL10 transcriptional repression and subsequent suppression of NK cells migration. Except for regulations in CXCL9/CXCL10, histone modification also controls immunological functions of TILs through various mechanisms, for example, Forkhead box P3 (Foxp3), a master lineage-specific phenotypic protein of Treg cells, was proven to play a new role in the TME by combined with HAT1 to control CC chemokine receptor 4 (CCR4) expression via providing provocative (K23 and K27) or repressive (K14 and K18) acetylation to H3, thus influencing the infiltration of Treg cells and anti-tumor immunity [221]. Moreover, the expression of Foxp3 itself was stabilized by acetylated histones at its promoter [222], indicating that histone epigenetic regulation has broad utility for modulating immune responses in TME.

The activation and function of TCLs is typically modulated by exosomes or EVs enriched with miRNA released from malignant cells. For instance, TNF- and CD45-targeting miRNAs such as miR-498, miR-181a/b, and miR-3187-3p could be released from melanoma cells to suppress the immunological function of CD8^+^ T cells [223], while delivering miR-142-5p from cervical squamous cell carcinoma (CSCC) cells to lymphatic endothelial cells (LECs) was considered as an integral component of the immune checkpoint, which could suppress and exhaust CD8^+^ T cells through AT-rich interactive domain-containing protein 2 (ARID2)-DNMT1-IFN-γ signaling and the elevated expression of indoleamine 2,3-dioxygenase (IDO) in the downstream [224]. What’s more, Zhou, J., et al. [225] found that TAM could also secrete exosomes contained miRNA including miR-29a-3p and miR-21-5p, which could be transfected into CD4^+^ T cells, leading to the direct suppression of STAT3 and an imbalance in Treg/Th17 cells, generating an immune-suppressive TME and associated with poor prognosis of Epithelial Ovarian Cancer (EOC) patients. The miR-29 is also identified being downregulated in NK cells in patients with T-cell acute lymphoblastic leukemia (T-ALL), with a reduction in IFN-γ production as well as NK group 2D (NKG2D) expression, who is one of the major activating receptors of NK cells binding to its ligands that are expressed on malignant cells, accompanied by accelerated ALL progression and decreased survival time [226, 227]. In conclusion, biological functions of miRNAs and their potential roles in modulating the differentiation, proliferation and activation of TLCs in TME along with remodeling immune homeostasis may profoundly influence tumor biology, as well as being potentially explored as therapeutic targets.

### Epigenetic modulation in other components in TME

Except for the epigenetic regulation towards multiple malignant cells and non-neoplastic cells in TME mentioned above, numerous other components, conjointly with their dynamic interrelationships within TME, substantially participate in tumorigenic processes and immunotherapy prognosis. Here, we primarily focus and draw brief conclusions on B lymphocytes cells and cancer stem cells (CSC).

Being one of the most decisive members in adaptive immune cells, B cells are majorly distributed at the infiltrative boundaries of tumors and the lymphatic structures near TME. Presenting a dual role in tumor biology [228], B cells produce antibodies to directly kill tumor cells and generate pro-tumorigenic cytokines to activate immunosuppressive cells [229], whose abundance has been confirmed to predict disease progression and patient survival [230, 231]. Although limited research focus on epigenetic modification towards B cells in TME, several evidence proved that the differentiation and malignance of B cells partially controlled by epigenetics. As an example, engaged in the pathogenesis of Burkitt lymphoma (BL), Epstein-Barr virus (EBV) infection would lead to the considerably suppression of histone methylation including H3K9me3 and H3K27me3, following the elevated transcriptional competence of most genomic regions, which might possibly explain the active immune response triggered by B cells [232]. Similarly, represented by mutation of the EZH2 protein, epigenetic disorders have been widely observed in acute lymphoblastic leukemia (ALL), non-Hodgkin lymphomas (NHLs) and various cancers originating from B cells malignances [233], while epigenetic mechanisms further participant in the high variability of CD20, the general surface marker expressed by the majority of B cells, which have been broadly demonstrated by treating different malignant B lymphocytes with either DNA methyltransferase inhibitor (DNMTis) or histone deacetylase inhibitors (HDACis) [234–236]. Further investigation in the pathology and immune function altered by epigenetics towards B cells in various tumors is needed.

During the past decades, the profound role of CSCs in tumor initiation, progression and regression has become the hottest topic in the field of oncology, and epigenetics has been esteemed as the key mechanism. Characterized as self-renewal and multi-directional differentiation capacity [237], CSCs are a subset of small cells owning the potential to strongly induce tumorigenesis and reproduce cells types in cancers [238]. There are compelling evidence verifying that epigenetic regulations play an extremely crucial role in governing the stemness and self-renew ability of CSCs, along with the abundance and distribution of which in TME. For example, DNA demethylation has been certified as the vital factor to CSCs generation, differentiation and pluripotency [239–242], and histone modifications enzymes such as EZE2 and HDACs have extensively involved in regulating key signaling in CSCs, aberrantly activating Wnt signaling pathway [243, 244], Hedgehog (Hh) signaling pathway [245] and Notch signaling pathway [239, 246]. Furthermore, thorough explorations into miRNAs have revealed the complicated effect they have on tumor biology including sphere formation and chemoresistance. For example, miR-519d stimulated mitochondrial apoptotic pathway therefore enhanced the therapeutic effect of chemotherapy drug cisplatin [247], while ectopic miR-181b equivalently sensitized cisplatin-resistance cancers by targeting to Notch2 signaling pathway [248]. Diverse epigenetic mechanisms modifying the characteristics and functions of CSCs attributed to the oncogenesis in different cancers have been exhaustively explained and concluded in numerous reviews [238, 249–251].

## Clinic trials and drugs targets epigenetic modifications in TME

**Fig. 3:**
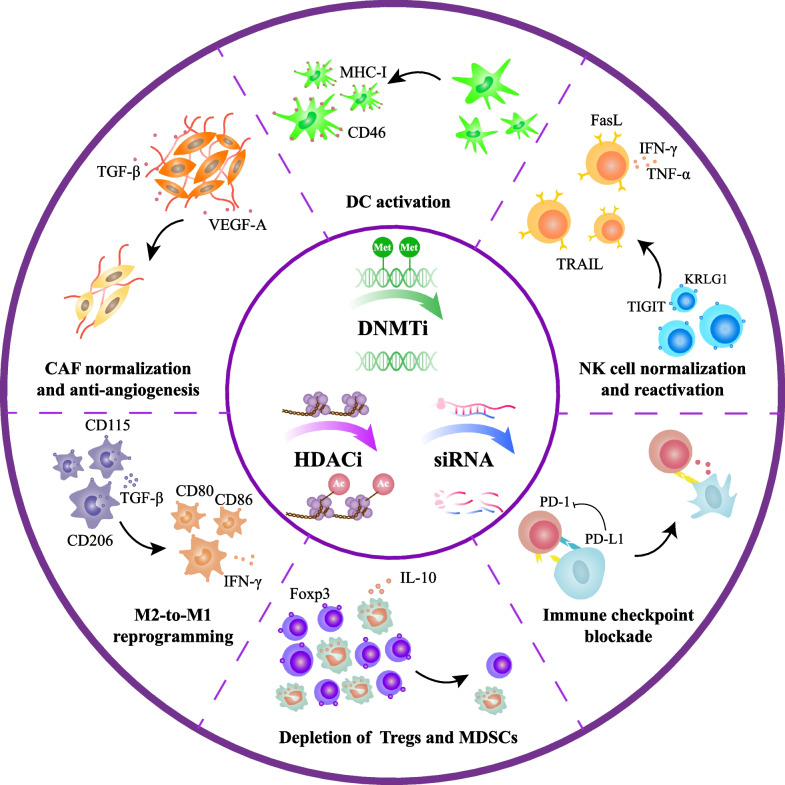
Pharmacological effects of epi-drugs on the components in TME. The inner circle represents basic pharmacological mechanisms of DNMTi, HDACi and siRNA. In brief, the former two epi-drugs inhibit essential enzymes (DNMT and HDAC) for the maintenance of aberrant epigenetic landscapes (DNA methylation and histone deacetylation), while the latter one directly binds to the target mRNA and blocks protein translation. Those effects eventually alter the tumor-favoring expression profiles in TME, which include but not limit to: 1) normalizing CAFs and anti-angiogenesis, 2) promoting antigen presenting capacity of DCs, 3) reversing the aberrant metastasis-promoting characteristics of NK cells and enhancing their antigen-dependent cell-mediated cytotoxicity, 4) blocking PD-L1, PD-1 and CTAL-4 to exploit the full anti-tumor potency of effector CD8 + T cells, 5) facilitating M2-to-M1 reprogramming process in TAMs, and 6) suppressing the recruitment of Tregs and MDSCs in TMEs while activating their inflammatory characteristics

Epigenetic (Epi)-drugs generally refer to artificial compounds that reverse the tumor-favoring epigenetic landscape in the molecular level, mainly by suppressing the essential enzymes for its establishment and maintenance [252]. Those inhibitors include DNMTis, HDACis, histone methyltransferase inhibitor (HMTis) and so on. Of note, some ncRNA-based drugs, especially small interfering RNAs (siRNAs), which refer to the 20–25-base-pairs oligonucleotides capable of arousing the degradation of mRNAs and subsequent gene silence [253], can directly involve in epigenetically regulating the expression level of target genes. Therefore, the siRNA-based drugs were also discussed as an exogenous epigenetic regulator that has therapeutic potential in cancer management (Fig. 3). Emerging epi-drugs have demonstrated inspiring therapeutic efficacy in hematological malignancies. However, most epi-drugs failed to achieve satisfying clinical outcomes in solid tumors due to blocked drug delivery caused by ECM deposition and competitive absorption by other components in TME. In contrast, the stromal cells and infiltrated immune cells in TME are directly exposed to the tissue fluid, making them more susceptible to the effect of epi-drugs. Therefore, TME components would be a promising target for epi-drugs in clinical cancer management (Table 2).

**Table 2:** Epigenetic therapies target TME

Class	Drug	Target	Mechanism	Cancer	Phase	Reference
CAFs /HSCs						
DNMTi	DAC	PTEN	PTEN ↑- normalize HSCs	HCC^a^	preclinical	[254]
	DAC + Eugenol	DNMT1/DNMT3A	DNMT1/DNMT3A↓- normalize CAFs	BC	preclinical	[255]
	5-AZA + Ruxolitinib	SHP-1	SHP-1↑-normalize CAFs	LC&HNSCC	preclinical	[133]
	DAC	DNMT3B	block the TGF-β1/miR-200 s/miR-221/DNMT3B loop	BC	preclinical	[137]
	DAC	SOCS1	SOCS1↑-STAT3 ↓-IGF-1 ↓	PDAC	preclinical	[256]
	DAC + Eugenol	VEGF-A and IL-8	VEGF-A↓, IL-8↓, suppress angiogenic factors	BC	preclinical	[257]
HDACi	CUDC—907	HDACs/PI3K	PI3K/AKT↓- TGF-β1↓	LC	preclinical	[142]
Endothelial cells						
HDACi	entinostat	HDAC1/2/3	SERPINF1↑, THBS2↑, PTEN ↑and p21↑- reduced VM structures	TNBC	preclinical	[258]
siRNA	ALN-VSP02	KSP and VEGF	KSP, VEGF↓- microvessel density ↓and vascular leakage↓	EC	stage II, NCT01158079	[259]
	LOx/CAT + siVEGF	VEGF	VEGF ↓and lactate ↓-suppress cancer migration	TNBC	preclinical	[260]
	peptide carrier L1 + siVEGFA + siVEGFR1	VEGFA, VEGFR1	VEGFA↓ and VEGFR1 ↓- anti-angiogenesis	BC	preclinical	[261]
	siVDAC	VDAC	VDAC1 ↓- TME-related genes—affect ECM-tumor crosstalk	LC	preclinical	[262]
	Atu027	PKN3	PKN3↓- tumor metastasis↓	PDAC	stage I, NCT00938574 stage II, NCT01808638	[263, 264]
	siLPP	LPP	LPP↓- microvessel leakiness↓	OC	preclinical	[265]
	siCXCL11	CXCL11	CXCL11↓- reverse SASP -cancer migration↓	BC	preclinical	[266]
NK cells						
DNMTi	DAC and 5-AZA	TIGIT and KLRG1	TIGIT↓ and KLRG1↓- normalize teNK cells	BC	preclinical	[267]
siRNA	LIP-NPs + siSHP-1 + siCbl-b + sic-Cbl	SHP-1, Cbl-b, and c-Cbl	unleash NK cell activity to eliminate tumors	\	preclinical	[268]
DCs						
DNMTi	5-AZA	CD40 and CD86	CD40 ↑and CD86↑; IL-10 and IL-27 secretion↓	\	preclinical	[269]
	5-AZA (A) and romidepsin (R), along with IFNα2 (ARI)	HDACs	synergistically endowed DCs with a marked migratory capability	CC	preclinical	[270]
HDACi	DOT1Li	H3K79	FOXM1↓, IL-12↑, promote BMDC maturation	PDAC/CC	preclinical	[271]
siRNA	DC vaccine + siIDO	Indoleamine 2,3-DiOxygenase (IDO)	silence expression of immunosuppressive enzyme IDO in DCs	BC	preclinical	[272]
TAMs						
DNMTi	5-AZA	IL-6&p65	p65 phosphorylation, IL-6 ↑, → M1-like phenotype	\	preclinical	[273]
siRNA	M2-like TAM dual-targeting NPs + siCSF1R	CSF1R	CSF1R↓, block the survival signal of M2-like TAMs	melanoma	preclinical	[274]
	PEG = MT/PC NPs + siVEGF + siPIGF	VEGF, PIGF	VEGF↓ and PIGF↓, promote M2-to-M1 reprogramming	BC	preclinical	[275]
	pH-sheddable PEG corona + siSTAT6 + inhibitor AS1517499	IKKβ	IKKβ ↓and STAT6 signaling ↓- M2-to-M1 repolarization	\	preclinical	[276]
	CL4H6-LNPs + siSTAT3	STAT3/HIF-1α	STAT3↓, M2-to-M1 repolarization	\	preclinical	[277]
Treg cells						
HDACi	entinostat	STAT3	STAT3↓- FOXP3↓	RC/PC	preclinical	[278]
	CPI-1205	EZH2	endow Treg cell with pro-inflammatory functions	\	preclinical	[123, 216, 279]
	Dznep	EZH2	depleted LMP1-HONE1 antigen-induced Tregs	NPC	preclinical	[280]
	PLX51107	EZH2	altered expression pattern similar to Foxp3-deficient Treg cells	melanoma	preclinical	[281, 282]
MDSCs						
DNMTi	DAC	IRF8	IRF8↑- MDSC accumulation↓	\	preclinical	[85]
HDACi	HDAC11i	HDAC11	decrease IL-10 expression, inhibit MDSC maturation		preclinical	[159]
	SAHA	(HDACs)	endogenous ROS↑, induce apoptosis of Gr1 + MDSCs	BC	preclinical	[283]
	CG-745	(HDACs)	IL-2 and IFN-γ ↑, suppresses M2 macrophages and MDSCs content	CC	preclinical	[284]
	trichostatin-A	(HDACs)	inhibit the recruitment of MDSCs	\	preclinical	[285]
	GNE-781	CBP/EP300-BRD-H3K27	Arg1 and iNOS↓- redirects MDSCs to an inflammatory phenotype	\	preclinical	[286]
DNMTi & HDACi	5-AZA + HDACi + α-PD-1	(DNMTs & HDACs )	reduced MDSCs through type I IFN signaling	OC	preclinical	[287]
effector CD8^+^ T cells						
HDACi	PLX51107	PD1	PD1↓, enhance ICI effects	melanoma	preclinical	[282]
siRNA	polyethylene glycol-chitosan-alginate (PCA) NP + siRNA	CTLA	block A2AR and CTLA-4, enhance antitumor responses of CD8^+^ T cells	\	preclinical	[288]
	siRNA mPEG-PLA-PHis-ss-PEI polyplexes + PD-L1 siRNA	PD-L1	promote CD8 + and CD4 + T cells infiltration	\	preclinical	[289]
	FX@HPs + PD-L1 siRNA	PD-L1	FX@HP induced T cell infiltration, increased calreticulin on tumor cells, and reduced MDSCs/Tregs	orthotopic lung tumors	preclinical	[290]
	indoleamine 2,3-dioxygenase inhibitor + PD-L1 siRNA	PD-L1	favor the survival and activation of cytotoxic T lymphocytes	BC	preclinical	[291]
	hyaluronic acid conjugate + PD-L1 siRNA	PD-L1	potentiate tumor-reactive T cells by blocking PD-L1	\	preclinical	[292]
m6A editing	CS1/CS2	FTO	suppress immune checkpoint genes, especially LILRB4, elevate T cell cytotoxicity	AML	preclinical	[186]
	ALK-04	ALKBH5	modulates Mct4/Slc16a3 expression and lactate content in TME, suppress Treg cells and MDSCs	melanoma	preclinical	[125]

^a^*HCC* hepatocellular carcinoma, *BC* breast carcinoma, *LC* lung carcinoma, *HNSCC* head and neck squamous cell carcinoma, *PDAC* pancreatic duct adenocarcinoma, *TNBC* triple negative breast carcinoma, *EC* endometrial carcinoma, *OC* ovarian carcinoma, *CC* colorectal carcinoma, *RC* renal cell carcinoma, *PC* prostate carcinoma, *NPC* nasopharyngeal carcinoma, *AML* acute myeloid leukemia

### Epi-drugs normalize CAF phenotype in TME

CAFs in TME often exhibit pro-tumorigenic characteristics due to tumor-induced phenotype reprogramming [293]. Such a process is highly dynamic and plastic that various anti-tumor drugs could easily affect it. Compared with cancer cells, the genomic landscape of CAFs is majorly reprogrammed by epigenetic modification rather than nucleotide mutation, thereby demonstrating higher responsiveness to epi-drugs [8]. Here, we summarized the existing epi-drugs that target CAFs in TME.

Many epi-drugs can directly normalize CAFs by reversing the tumor-induced epigenetic alterations. Among them, DNMTis (including 5-azacitidine, decitabine and eugenol) demonstrated robust therapeutic potential in remodeling the DNA methylation landscape of CAF-associated genes to promote CAF normalization. In 2012, Bian et al. first reported that decitabine could significantly decrease hypermethylation of the phosphatase and tensin homolog (PTEN) promoter in hepatic stellate cells (HSCs) and thus reversed fibrosis-promoting phenotypes by rescuing PTEN expression [254]. Since HSC-mediated fibrosis plays a crucial role in tumor progression and drug resistance of HCC, decitabine is a promising agent to enhance the efficacy of systematic treatment for advanced HCC. Similarly, Al-Kharashi et al. observed that decitabine and eugenol downregulated DNMT1 and DNMT3A through E2F1-mediated pathway [255], which blocked VEGF-A and IL-8 secretion and reversed the pro-angiogenic phenotype of CAFs in breast cancer [257]. Besides, Xiao et al. discovered that decitabine could rescue PDAC-induced methylation of a cytokine signaling suppressor termed suppressor of cytokine signaling 1 (SOCS1) in CAFs, thereby decreasing the secretion of insulin-like growth factor-1 (IGF-1) through JAK1/STAT3 pathway [256]. Intriguingly, combining 5-AZA with ruxolitinib (a JAK1/STAT3 inhibitor) could synergistically abrogate JAK1/STAT3 pathway in CAFs and potentiate CAF normalization in LC and HNSCC. Mechanistically, 5-AZA reversed the hypermethylation state of src homology 2 domain-containing protein tyrosine phosphatase 1 (SHP-1) promoter and increased the expression of endogenous JAK1/STAT3 inhibitor SHP-1, while ruxolitinib suppressed JAK1-induced phosphorylation of STAT3 [133]. Of note, a very recent study reported that DNMT3A could downregulate grainyhead like transcription factor 2 (GRHL2) and cadherin 1 (CDH1) while upregulated zinc finger E-box binding homeobox 1 (ZEB1) and vimentin (VIM) in PC3 cells, promoting CAF-induced epithelial-mesenchymal transition (EMT) and resulting in worse prognosis [294]. Since EMT is a hallmark of castration resistance, cancer stem cells generation, chemoresistance and worse prognosis for prostate cancer, targeting DNMT3A may be of great therapeutic value in improving the prognosis of advanced and metastatic prostate cancer.

Some DNMTis and HDACis could desensitize fibroblasts to CAF-promoting signaling such as TGF-β, thus blocking CAF maturation in TME. For example, a first-in-class HDACi termed CUDC-907 can suppress TGF-β1-induced CAF proliferation as a dual inhibitor of HDACs and PI3K/AKT pathway [142]. Intriguingly, Tang et al. discovered a positive feedback loop that facilitated TGF-β autocrine in breast cancer, where TGF-β1 could activate DNMT3B and thus suppressed the expression of miR-200a, miR-200b, miR-200c and miR-141, leading to enhanced sensitivity of CAFs to TGF-β1 [137]. Exposure of CAFs to decitabine significantly blocked TGF-β1 autocrine in CAFs and thereby normalized CAFs in breast cancer.

SiRNA-based drugs could precisely silence a specific CAF-driving gene and suppress an indicated pro-tumorigenic function. For instance, silencing IL11 and IL15 in CAFs by siRNAs inactivated STAT3 signaling and repressed CAF-induced angiogenesis in breast cancer [295]. In addition, the silence of siRNA-mediated yes1 associated transcriptional regulator (YAP1) in CAFs reduced the generation of collagen fibers and inhibited angiogenesis [296]. Of note, emerging evidence with regard to CAF heterogeneity indicated that certain subgroups of CAFs may serve as a double-edge sword rather than merely enhances tumor progression [293, 297]. In this context, precisely blocking their pro-tumor function by siRNA seems to be a better choice in CAF-targeting therapy.

### Epi-drugs suppress neovascularization in TME

Tumor neovascularization is critical for sustained tumor growth and metastasis. Those immature vessel-like structures lead to hyperpermeability, poor perfusion, and hypoxia that further accelerates tumor progression and immune evasion. As mentioned above, DNMTis could refrain the secretion of multiple pro-angiogenetic cytokines such as VEGF-A and IL-8 [257]. However, Hegde et al. found that DNMT1 could inhibit IL-6-induced VEGFR2 upregulation in endothelial cells in breast cancer, suggesting that DNMTi may have a side effect on promoting angiogenesis [298]. For HDACis, a class I HDAC inhibitor termed entinostat could epigenetically activate anti‑angiogenic genes including serpin family F member 1 (SERPINF1) and thrombospondin 2(THBS2) and reduced the formation of vasculogenic mimicry (VM) in triple negative breast cancer, thereby inhibiting tumor metastasis and improving the overall survival of the murine breast cancer models [258].

Notably, siRNA-based drugs are essentially efficient in repressing angiogenesis. A phase II trial demonstrated that ALN-VSP02, an LNP-formulated siRNA drug targeting cadherin 16 (CDH16) and VEGF in neoplastic cells, could dramatically decrease microvessel density and vascular leakage in TME and worked well in repressing liver metastatic sites of EC [259]. Similarly, Tang et al. used the cationic liposomes to deliver the cascaded enzymes lactate oxidase/catalase and siVEGF, which both depleted lactate accumulation and silenced VEGF expression and exerted essential anti-angiogenetic effect for triple negative breast cancer [260]. Moreover, dual silencing of VEGFA in cancer cells and VEGFR1 in endothelial cells significantly suppressed VM formation in breast cancer, suggesting that codelivery of multiple siRNAs is a promising strategy to improve the anti-angiogenetic effects [261]. Intriguingly, a very recent study reported that silencing voltage-dependent anion-selective channel protein 1 (VDAC1) in neoplastic cells could not only inhibit tumor-mediated angiogenesis, but has an overall impact on a battery of TME-associated genes involved in extracellular matrix construction, and intercellular interaction in LC, highlighting the therapeutic potential of siVDAC1 in stromal-abundant solid tumors [262].

Endothelial cells in TME can also acquire tumor-promoting characteristics by epigenetic reprogramming. In this context, siRNA-based drugs demonstrated great efficacy in reversing the aberrant phenotype of endothelial cells. For example, microvessel leakiness is a major cause for drug delivery failure and chemotherapeutic resistance. Leung et al. discovered that silencing LIM domain containing preferred translocation partner (LPP) decreased microvessel leakiness and improved paclitaxel delivery to neoplastic cells, which dramatically improved chemotherapeutic efficacy of paclitaxel in ovarian cancer [265]. Notably, endothelial cells could facilitate tumor metastasis by expressing tumor adhesion ligands. An siRNA drug called Atu027 silenced the expression of a pro-metastatic ligand termed protein kinase N3 (PKN3) in endothelial cells and induced dramatic regression of distant metastasis of pancreatic cancer in phase I and II clinical trials [263, 264]. A very recent study found that the senescence associated secretory phenotype (SASP) of endothelial cells could promote tumor metastasis. SASP inhibition by silencing CXCL11 could significantly attenuate the migration and spheroid invasion of MDA-MB-231 cells in vivo [266].

Taken together, siRNA-based drugs displayed great advantages in suppressing angiogenesis and pro-tumorigenic phenotype of endothelial cells. Of note, synergistic therapy combining siRNAs with other sorts of epi-drugs may serve as a promising strategy to improve the clinical outcomes. However, there is still a long way to go in the development of siRNA-based drugs because most of the carrier particles fail to combine low cytotoxicity and high delivering efficacy simultaneously. Developing bionic nanoparticles with cell-specific ligands would contribute to overcoming the dilemma in clinical practice.

### Epi-drugs repress the immunosuppressive components in TME

Immunosuppressive cell populations are widely distributed in TME, including MDSCs, Tregs and regulatory B cells (Bregs). Inborn with the capability to repress anti-tumor immunity, those immunosuppressive components dramatically impair the clinical efficacy of immunotherapy. One way to eliminate the infiltrated MDSCs in TME is apoptosis induced by epi-drugs. For example, decitabine decreased MDSC accumulation by disrupting DNA methylation of RIP1-dependent necroptotic genes and facilitating MDSC elimination in TME [85]. An HDACi called SAHA could induce apoptosis of Gr1^+^ MDSCs in breast cancer by increasing endogenous ROS in MDSCs [283]. Another HDACi called CG-745 increased IL-2 and IFN-γ, which could suppress M2 macrophages and MDSCs content [284]. Besides, epigenetic inhibition of immunosuppressive biomarkers can serve as another strategy to remodel the immunosuppressive characteristics of MDSCs. For instance, an HDACi called GNE-781 inhibited CBP/EP300-BRD and transformed MDSCs to an inflammatory phenotype by inactivating STAT pathway and repressing Arg1 and iNOS [286]. Intriguingly, a triple combination of 5-AZA, HDACi and α-PD-1 increased the infiltration of CD45^+^ immune cells, NK cells and active CD8^+^ T cells, while reducing tumor burden and extending survival through type I IFN signaling, while triple combination provided the best anti-tumor performance in the murine models [287]. However, Sahakian et al. discovered that, in contrast to their wild-type counterparts, tumor-bearing HDAC11-knockout mice displayed increased IL-10 expression and a more suppressive MDSC population [159]. In addition, another HDACi called trichostatin-A was also found to exert dual effects on tumor immunity. On one hand, trichostatin-A modulated the suppressive activity of infiltrating macrophages and inhibited the recruitment of MDSCs. On the other hand, trichostatin-A also upregulated PD-L1 expression on malignant cells, thereby limiting the beneficial therapeutic effects [285].

Unlike the infiltrated MDSCs migrating from other sites, most Treg cells in TME derive in situ due to tumor-mediated intentional reprogramming. In this context, epi-drugs could remarkably contribute to inhibiting Treg maturation and acquirement of immunosuppressive biomarkers [299]. For example, entinostat could reduce STAT3 acetylation by inhibiting HDAC1/3, leading to corollary repression of Foxp3 in Treg cells in renal and prostate cancer [278]. In addition, an EZH2-targeting HDACi called CPI-1205 drove the acquisition of pro-inflammatory functions of Treg cells in the TME, with a coordinate promotion in the recruitment of CD8^+^ and CD4^+^ effector T cells [123, 216, 279]. Similarly, another EZH2 inhibitor named Dznep depleted LMP1-HONE1 antigen-induced Tregs in vitro and led to promoting TAA-specific antitumor response in nasopharyngeal cancer [280]. Of note, a novel HDACi PLX51107 (inactivating the histone acetylated protein BRD4) was found to induce EZH2 deficiency in Treg cells. Surprisingly, PLX51107-treated Treg cells in vitro demonstrated a similar gene landscape to Foxp3-deficient Treg cells that lost the immunosuppressive function in melanoma [281, 282]. Compared with HDACis, DNMTis appeared to exert dual function in inhibiting Treg cell derivation in TME. A DNA demethylase ten-eleven translocation (TET) enzyme was found to increase the stability of Foxp3 expression in TGF-β-induced Treg cells through Tet2/Tet3 signaling, suggesting the possible application of DNMTis in repressing Treg cells [300]. However, a very recent study discovered that decitabine could increase Treg infiltration during HBV infection, probably by promoting Treg differentiation from naïve CD4^+^ T cells [301]. In additional, siRNA-based drugs could precisely block the Treg-activating ligands in TME. Knocking down the expression of membrane-associated phosphatidylinositol transfer protein 3 (PITPNM3) significantly reduced CCL18 recognition in intertumoral Tregs, which subsequently inhibited naive CD4^+^ T cell recruitment and enhanced immunotherapeutic efficacy of anti-PD-1 antibodies [302].

### Epi-drugs facilitate M2-to-M1 repolarization of TAMs

As the pivotal orchestrator in TME, the functional status of TAMs could largely determine the activity of anti-tumor immune response [303]. In this context, M2-to-M1 reprogramming emerges as a robust way to reverse the immunosuppressive landscape of TME due to high plasticity of TAM phenotype in TME. Several epi-drugs have been reported to inhibit the M2-to-M1 repolarization in TME. Shi et al. reported that 5-AZA could epigenetically increase IL-6 expression and p65 phosphorylation, thus stimulating the activation of macrophages toward an M1-like phenotype and subsequent T cell recruitment in TME [273]. Intriguingly, siRNA drugs encapsuled in functionalized nanoparticles (NPs) have attracted great attention in M2-to-M1 repolarization. Compared with nude siRNAs, NP-mediated siRNA delivery can remarkably prevent premature drug degradation, reduce side effects and improve drug absorption. Moreover, multifunctional NPs can be designed to affect the immune-activating pathways and metabolic pattern in TME, which further contributes to reprogram M2-like TAMs. For example, Qian et al. developed M2-like TAM dual-targeting NPs modified with α-peptide and M2 macrophage binding peptide. Loading anti-colony stimulating factor-1 receptor (CS1FR) siRNA into these NPs, they observed significant decrease of IL-10 and TGF-β production and subsequent M2-TAM depletion in melanoma [274]. Another lipid nanoparticle composed of a novel pH-sensitive cationic lipid CL4H6 (CL4H6-LNPs) was developed to deliver siSTAT3, which significantly increased the infiltration of M1 TAMs and reversed TAM-induced angiogenesis in TME [277].

In addition, some smart nanodrugs, which are endowed with specific responsiveness to physicochemical property of TME, exhibit higher specificity and precision in controlling siRNA release. For instance, Song et al. created polyethylene glycol and mannose doubly modified trimethyl chitosan modified with citraconic anhydride grafted poly-based nanoparticles (PEG = MT/PC NPs) with intelligent pH-responsiveness to deliver siVEGF and siPIGF (siRNA for placental growth factor). This strategy achieved efficient gene silencing, promoted M2-to-M1 reprogramming in TAMs, and exerted robust suppression of breast cancer proliferation and lung metastasis [275]. Similarly, Xiao et al. designed a smart micellar nanodrug with M2-targeting peptides hidden in the pH-sheddable PEG corona so that the contents would only be released in the acidic TME. These intelligent NPs could co-deliver siIKKβ and STAT6 inhibitor AS1517499 to reprogram M2-like TAMs, without affecting the normal tissues and causing adverse effects [276]. Taken together, siRNA-based nanodrugs could effectively and specifically target TAMs in TME with high biocompatibility and multifunctional integration, thereby being the most promising strategy for M2-to-M1 repolarization in clinical application.

### Epi-drugs reactivate DCs and NK cells in TME

DC vaccines are the newly emerging technique of tumor immunotherapy that boost antigen presentation in TME in order to exploit the full potential of the adaptive immune response to eliminate tumor cells [304]. Unfortunately, most DC vaccines failed to achieve satisfying clinical outcomes due to tumor-induced DC inactivation by epigenetic modification. Frikeche et al. observed that 5-AZA exposure significantly increased the expression of DC-promoting ligands CD40 and CD86 on mature DCs, and suppressed IL-10 and IL-27 secretion that impaired CD8^+^ T cytotoxicity [269]. Another study revealed that H3K79 demethylation by DOT1Li could reduce forkhead box M1 (FOXM1) expression and increase IL-12 production, thus promoting DCs maturation in breast cancer [271]. Intriguingly, Fragale et al.found that combinatory use of 5-AZA, romidepsin (an HDACi) and IFNα2 (ARI) remodeled the IFN signature and endowed DCs with a marked migratory capability in vivo. Those DCs actively participated in T cell cross-priming in tumor draining lymph nodes [270]. However, Liu et al. proposed an opposite perspective that DNMTi may impair the responsiveness of DCs to TLR3 or TLR4 signaling by increasing the expression level of TLR inhibitor SOCS1, abolishing polyinosinic-polycytidylic acid or LPS-induced DC maturation in TME [305]. Of note, siRNA pretreatment could dramatically potentiate the immunogenicity of DC vaccines, probably due to combinatory role where siRNAs both serve as the immunologic adjuvant and inhibit tumor-induced immunosuppressive phenotype of DCs. Pretreating DC vaccines with siIDO in vitro silenced expression of indoleamine 2,3-DiOxygenase (IDO, an immunosuppressive enzyme) and achieved better tumor-eliminating effects in the murine breast cancer models [272]. Since siRNA pretreatment in vitro could avoid the complex signaling interference in TME, it may serve as a robust strategy to unleash the anti-tumor potential of DC vaccines.

Similar to DCs, the anti-tumor activity of NK cells is often restricted in TME due to tumor-mediated epigenetic modification, whereas some epi-drugs could reactivate NK cells by suppressing NK-inhibiting gene expression. Chan et al. found that decitabine and 5-AZA could rescue NK cells from a metastasis-favoring phenotype in breast cancer by hypomethylation-induced repression of NK-inhibiting genes including T cell immunoreceptor with Ig and ITIM domains (TIGIT) and killer cell lectin like receptor G1 (KLRG1) [267]. In contrast, Kdm5a (an H3K4me3 demethylase) could resisted repressive chromatin configuration at the promoter of NK-activating cytokine suppressor SOCS1 and further impaired NK cell activity, whereas Kdm5a inhibitor re-silenced SOCS1 and rescued the NK-mediated immune response in TME [306]. In addition, siRNA-mediated silencing of the key intrinsic inhibitory NK cell molecules, including SHP-1, Cbl-b, and c-Cbl, could unleash NK cell activity to eliminate tumor cells, providing a new possibility of NK-targeting siRNA therapy [268]. Nevertheless, some epi-drugs like DNMTis and HDACi may have a negative impact on NK activation. 5-AZA and butyric acid (an HDACi) could increase the expression of NK-inactivating ligands called Siglec-7 on NK cells, thus inhibiting NK maturation in TME [307]. Besides, DNMTis could inhibit LPS-induced release of IL-1β, thereby impairing the production of NK-derived proinflammatory factors [308]. Further investigation should be conducted to evaluate the NK-activating effects by epi-drugs in clinical trials.

In summary, epi-drugs exhibit great potency in reinforcing the immunogenicity of DC vaccines and reactivating NK cells in TME, while siRNA-pretreated DC vaccines provide a novel insight into DC-based tumor immunotherapy. Of note, some DNMTis and HDACis appear to have dual effects on reactivating DC and NK cells, but the underlying mechanisms remain unknown. In this context, more efforts should be made to figure out the reason for such dual effects in order to avoid their immunosuppressive effects in clinical practice.

### Combining epi-drugs with immune checkpoint inhibitors

The CD8^+^ T effector cells play a vital role in selective eradicating cancer cells. Nevertheless, overexpression of immune checkpoints on tumor cells, such as PD-L1 and CTLA-4, impairs the tumor-killing potency of CD8^+^ T cells in TME. In this context, immune checkpoint inhibitors (ICIs) are developed to exploit their full potency of tumor eradication and have demonstrated inspiring clinical efficacy in advanced melanoma and several hematological malignancies [309]. But when applied to treat cancers with immunodeficient TME, ICIs often failed to induce a durable anti-tumor response due to epigenetically induced CD8^+^ T anergy. Recent clinical trials have revealed that ICIs' therapeutic efficacy could be significantly increased by combining epi-drugs and ICIs in a synergistic therapy. According to one theory, epi-drugs increase the exposure of tumor-associated antigens and upregulate the expression of immune-activating genes in CD8^+^ T cells, which would synergistically wake up the disabled CD8^+^ T cells and encourage tumor cleavage in TME [310].

DNMTis could serve as a beneficial partner to improve the clinical efficacy of ICIs. On the one hand, DNMTis could upregulate MHC I and II molecules expression in cancer cells, which increased tumor immunogenicity and stimulated the anti-tumor response in TME. On the other hand, DNMTis could also suppress the infiltration of immunosuppressive components in TME [311]. In 2019, a two-arm phase II study enrolling 86 patients with refractory classic Hodgkin lymphoma demonstrated that patients who received decitabine plus camrelizumab achieved 71% complete remission compared with 32% in camrelizumab monotherapy group. Meanwhile, decitabine significantly reversed resistance to PD-1 inhibitors in patients with refractory classic Hodgkin lymphoma [312]. Recently, a novel DNMTi called CM-272 was found to improve the efficacy of PD-1 inhibitors in advanced bladder cancer by suppressing the expression of G9a and inducing immunogenic cell death [313]. Intriguingly, Srour et al. discovered that protein arginine methyltransferase 7 (PRMT7)-deficient melanoma demonstrated lower level of DNA methylation in the endogenous retroviral elements (ERVs), which enhanced gene expression regarding antigen process, the IFN pathways and chemokine secretion in CD8^+^T cells. Combining PRMT7 inhibitors with ICI therapy induced a strong anti-tumor T cell immunity in TME and restrained tumor growth in vivo [314].

“HDACis + ICIs” has been the most extensively studied combinatory strategy. Similar to DNMTis, HDACis upregulate the expression of TAP-1 and TAP-2 which participate in the intracellular transportation of tumor-associated antigen, thus enhancing the MHC-I presentation. In addition, HDACis could increase the secretion of IFN-α/β, and reverse T-cell exhaustion in TME. Furthermore, HDACis were found to decrease Foxp3 expression and impair suppressive function of Tregs [315]. In 2019, a phase I/Ib trial testing the combination of an anti-PD-1 antibody pembrolizumab and a pan-HDAC inhibitor vorinostat in 33 advanced NSCLC patients achieved 63% disease control rate and demonstrated preliminary antitumor activity in ICI-naïve patients [316]. Another phase Ib/II trial in 2021 also proved enhanced therapeutic efficacy of pembrolizumab in NSCLC patients with the assistance of the class I specific HDACi entinostat [317]. Similarly, the anti-PD-1 ICI nivolumab and the anti-PD-L1 ICI atezolizumab were also tested in combination with entinostat in several ongoing clinical trials, respectively (NCT02697630, NCT01928576, NCT02453620, NCT02708680) [318]. At present, an open-label phase I study is being conducted to explore the combinatory efficacy of HDAC6-specific inhibitor rocilinostat (ACY-241) with several anti-PD-1 and anti-CTLA4 ICIs in melanoma patients (NCT02935790) [319].

Except for clinical trials, a dual inhibitor for PI3K and HDAC termed BEBT-908 was developed to facilitate tumor ferroptosis by hyperacetylating p53 and upregulating the endogenous expression of MHC-I in tumor cells [320]. BEBT-908 significantly potentiated the effects of anti-PD1 ICIs and suppressed tumor growth in vivo. Qin et al. combined a specific inhibitor for histone lysine-specific demethylase (LSD1) with anti-PD1 ICIs, which significantly suppressed progression and pulmonary metastasis of breast tumor [321]. Similarly, Que et al. tested the combination of chidamide with an anti-PD-1 ICI toripalimab in metastatic sarcoma, which significantly promoted tumor regression and improved overall survival in the murine model [322]. Of note, Yang et al. developed a selective HDAC8 inhibitor which potentiated eradication of established hepatomas by anti-PD-L1 therapy, and the HSCC murine models receiving the combination therapy remained tumor-free for more than 15 months [323]. Another selective HDACi termed PLX51107 could also decrease the PD1 expression on CD8^+^ T cells, and enhanced therapeutic effects of ICIs in the murine melanoma models [282]. Interestingly, Tay et al. reported that HDAC3 could inhibit CD8^+^ T cell activation at the early stage, but was later required for persisting the activated status of CD8^+^ T cells, revealing the dual role of HDAC in regulating CD8^+^ T cell function [324]. However, it still remains unclear whether HDACis would impair the maturation of CD8^+^ T cells in TME.

Significantly, siRNA-based drugs demonstrated considerable efficiency in blocking the expression of immune checkpoints, suggesting potential as a novel immunotherapy strategy. For example, polyethylene glycol-chitosan-alginate NPs carrying anti-CTLA-4 siRNA could concomitantly block A2AR and CTLA-4 and facilitated CD8^+^ T-mediated tumor eradication in TME [288]. Another dual-responsive mPEG-PLA-Phis-ss-PEI polyplexes carrying anti-PD-L1 siRNA and resveratrol (a glycolysis inhibitor) promote CD8^+^ and CD4^+^ T cells infiltration by enhancing IFN-γ secretion and reprogramming the metabolic pattern in TME [289]. Moreover, FX@HP nanocomplex composed of fluorinated polymerized CXCR4 antagonism (FX) and paclitaxel-loaded human serum albumin (HP) could increase calreticulin on orthotopic lung tumor cells, which could work synergistically with the encapsuled anti-PD-L1 siRNA to facilitate T cell infiltration [290]. Based on the homing and penetrating peptides of breast tumor, a peptide assembling nano-delivery system was developed to deliver siPD-L1 and indoleamine 2,3-dioxygenase inhibitor in order to activate cytotoxic CD8^+^ T cells, and ultimately enhanced ICI-induced apoptosis of breast cancer cells [291]. A very recent study reported a polymeric nanoconjugate with innate immunogenicity, consisting of siPD-L1-based polyplexes, PEGylated hyaluronic acid as the CD44-targeting moiety, and ovalbumin as a model foreign antigen. Using such an immunostimulatory NP to carry siPD-L1 can remarkably potentiate DCs and tumor-reactive T cells [292].To draw a brief conclusion, DNMTis and HDACis could affect the expression landscape of immunoregulatory genes and target different immune cell population at the same time (Table 3). Rather than exerting catch-all effects, siRNA-based drugs are more precise and efficient in silencing the expression of immune checkpoints without excessive adverse effects. Notably, since tumor-induced epigenetic remodeling of immune cells plays a vital part in immunotherapeutic resistance, combining epi-drugs with ICIs could serve as a robust weapon to resist tolerance and improve clinical efficacy, representing as a promising trend in clinical immunotherapy.

**Table 3:** Clinical trials combining epi-drugs and ICIs

Class	Epi-drug	ICI	Disease	Phase	Record	State
DNMTi	decitabine	camrelizumab	refractory classic Hodgkin lymphoma	II	NCT02961101, NCT03250962	Completed
HDACi	vorinostat	pembrolizumab	non-small cell lung cancer	I/Ib	\^a^	Completed
HDACi	entinostat	pembrolizumab	non-small cell lung cancer	Ib/II	NCT02437136	Completed
HDACi	entinostat	pembrolizumab	metastatic uveal melanoma	II	NCT02697630	Completed
HDACi/DNMTi	azacitidine/entinostat	nivolumab	non-small cell lung cancer	II	NCT01928576	Not recruiting
HDACi	entinostat	ipilimumab/ nivolumab	HER2-negative breast cancer	I	NCT02453620	Not recruiting
HDACi	entinostat	atezolizumab	triple negative breast cancer	Ib/II	NCT02708680	Completed
HDACi	ACY-241	ipilimumab/ nivolumab	Melanoma	I	NCT02935790	Completed

^a^ Record number haven’t been found yet [316]

### Enhance the immune response via indirect mechanisms

Despite remodeling of the expression profile of immunoregulatory genes, some epigenetic drugs can indirectly affect tumor immunity via other mechanisms. One way is to induce type I interferon (IFN-I) response in TME. IFN-I response refers to a classic pro-inflammatory response that mobilizes the innate and adaptive immune cells to fight against infections and tumors. In details, various immune cells can produce the IFN-I superfamily ligands that specifically bind to a heterodimeric receptor comprised of IFNAR1 and IFNAR2, activate JAK/STAT pathway and ultimately initiate the transcription of IFN-inducible genes [325]. In TME, IFN-I response plays a crucial role in modulating cell growth, cell differentiation and tumor immune surveillance [326]. Moreover, recent studies have highlighted the potent anti-metastatic properties of IFN-I response in multiple sorts of solid tumors [325]. Intriguingly, epi-drugs such as DNMTis and siRNAs could initiate an unintended IFN-I response in TME through various mechanisms. Chiappinelli et al. reported that DNMTis induced cytosolic sensing of double-stranded RNA and caused IFN-I response in ovarian cancer [327]. In details, DNMTis reversed hypermethylated ERVs in neoplastic cells, which further activated dsRNA sensors TLR3 and melanoma differentiation-associated protein 5 (MDA5) and stimulated IFN-I response in TME. Furthermore, overexpression of ERVs in tumors significantly associates with durable clinical response of ICIs in melanoma patients. Another DNMTi named zebularine specifically sensitized the cGAS-STING pathway in response to DNA stimulation and upregulated interferon-stimulated genes, promoting infiltration of CD8^+^ T cells and NK cells in TME [328]. For most siRNAs, they often induce an unintended IFN-I response in which the exogenic siRNAs activate the dsRNA-dependent protein kinase termed protein kinase R (PKR) and upregulate IFN-β secretion [329]. Besides, siRNAs could be recognized by TLR8 in immune cells, triggering a TLR-mediated inflammatory response in TME [329, 330]. Nevertheless, since siRNAs are recognized as a foreign antigen in IFN-I response, whether the IFN-I response would attenuate the therapeutic effects of siRNA-based drugs still requires further investigation.

Despite the huge mutation burden carried by tumor cells, few tumors exhibit strong immunogenicity due to the impaired antigen processing system, such as silencing of MHC-I related genes, inactivated antigen-processing cells and decreased expression of costimulatory receptors [331]. Loss of tumor immunogenicity represents one of the core mechanisms in the initiation of immune evasion and immunotherapeutic resistance, posing a great challenge to improve tumor immunotherapy in clinical practice [332]. In this context, some epi-drugs were identified to improve tumor immunogenicity by increasing the expression of MHC-I and other costimulatory receptors on tumor cells. Pharmacological DNMT and HDAC inhibition reversed the repressive chromatin states of HLA-I genes, thereby increasing HLA-I expression on prostate cancer cells and making it more visible to immune surveillance [333]. In addition, HDACis such as Trichostatin A and VPA increased expression of antigen-processing-associated genes (TAP, LMP, tapasin genes and MHC I) on melanoma cells and other malignancies [334, 335]. Of note, a dual inhibitor of HDACs and DNMTs called CM-272 increased therapeutic efficacy of TCR-mediated DC vaccination, which further improved overall survival of B16-OVA tumor-bearing mice [205]. Similarly, decitabine and two HDACis (valproic acid and suberoylanilide hydroxamic acid) coordinately increased tumor antigen expression in malignant pleural mesothelioma and facilitated the therapeutic efficiency of ICIs [336].

Intriguingly, HDACis could enhance NK cytotoxicity by increasing expression of ligands required for the antibody-dependent cellular cytotoxicity (ADCC) on tumor cells. For example, CUDC-907 (a dual HDAC and PI3K inhibitor) caused dramatic regression of chemo-resistant multiple myeloma cell lines by upregulating natural killer group 2D (NKG2D) ligands and enhancing the ADCC effects in multiple myeloma cells [337]. A pan-HDAC inhibitor called panobinostat increased the expression of genes associated with cell adhesion, which guaranteed the process of NK-tumor conjugation and contributed to the increased tumor cytolysis [338]. Cho et al. monitored the effects of six selective HDACis on the expression of NKG2D and MHC-I molecules in lung cancer cells, and discovered that HDAC1 and HDAC2 might be the key orchestrators of NKG2D expression in lung cancer [339]. Mechanistically, HDACis stretch the chromatin structure of the promoter region and increased the accessibility for interferon-induced protein with tetratricopeptide repeats 1 (IFIT1) gene. Consequently, the increased levels of IFIT1 augmented the IFIT1-mediated IRF1, STAT4, and STING pathways and promoted NK cell-mediated IFN-γ production and cytotoxicity [340].

Metabolism pattern of TME components has a profound impact on the activity and functional landscape of immune cells. In details, tumor-derived metabolites can involve in the immunoregulatory signaling crosstalk and remodel the functional state of immune cells [341]. Besides, metabolic competition in the tumor ecosystem limits nutrient availability of immune cells, leading to microenvironmental acidosis and the subsequent impairment of immune cell function [342]. Some epi-drugs are capable of tumor metabolic reprogramming to stimulate the anti-tumor immunity. For example, tumor-derived cytokines could disrupt methionine metabolism in CD8^+^ T cells, causing methylation at H3K79me2 and impairing T cell immunity [88]. In contrast, blocking tumor SLC43A2 restored H3K79me2 in T cells, thereby boosting their innate potency of tumor eradication. In addition, m6A demethylation mediated by FTO elevated the transcription factors such as C/EBPβ, JunB and c-Jun, in neoplastic cells, which allowed the rewiring of glycolytic metabolism. Indeed, FTO inhibitor Dac51 impaired the glycolytic activity of neoplastic cells and indirectly restored the activity of CD8^+^ T cells [343].

## Outlook

Despite the fact that hundreds of studies have recently concentrated on the role of epigenetic regulation in a variety of tumors along with their microenvironment, revealing the profound influences on tumor initiation, metabolism, angiogenesis, immunosuppression and prognosis, leading to the development of numerous epi-drugs targeting some specific key molecules, further confirming the unique and distinct role of these epi-therapeutic modalities compared with traditional chemotherapy and radiotherapy, however, many questions are waiting to be resolved.

Recent years, increasing studies have uncovered that some ordinary genes regulating normal physiological activities or some non-coding genes silencing in normal tissues may possess potential epigenetic regulatory functions or may have abnormal epigenetic regulatory effects under malignant situations. Except for CLOCK, generally relevant to circadian rhythms but exhibited as the histone acetyltransferase in glioblastoma as mentioned before [127], Alvisi., et al. [344] lately manifested that in intrahepatic cholangiocarcinoma, the high enrichment of a novel transcription factor in tumor-infiltrating Tregs, mesenchyme homeobox 1 (MEOX1), was closely related to EZH2, further reprogram circulating Tregs. Although relevant research is temporarily limited right now, the conventional field of epigenetics may be greatly impacted by increased research on those potential genes, and the tumor landscape may benefit greatly from this innovation.

**Fig. 4:**
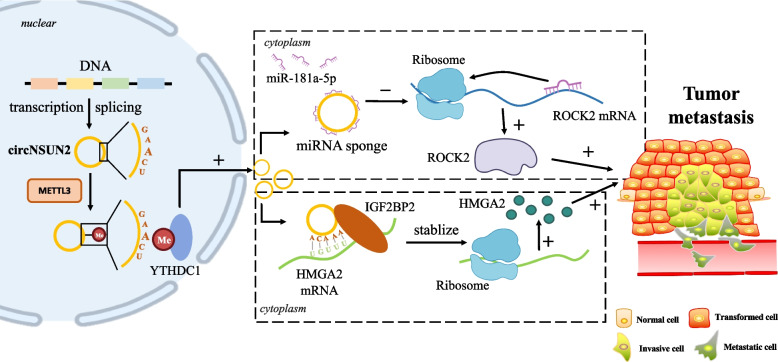
CircRNA (CircNSUN2) enhances the malignant biological behavior of CRC via two mechanisms. Catalyzed by METTLE3 and recognized by YTHDC1, circNSUN2 is exported from the nuclear to the cytoplasm in an m6A methylation-dependent manner. On the one hand, elevated cytoplasmic circNSUN2 prominently promotes the stability of HMGA2 mRNA via interaction with IGF2BP2, ultimately resulting in the aggressive nature of CRC cells. On the other hand, by sponging miR-181a-5p, circNSUN2 represses the miRNA blocking of ROCK translation, upregulates the expression of ROCK and leads to the increased proliferation and migration of CRC

In addition to the potential epigenetic targets, countless limitations in the traditional field of epigenetic regulation still need further breakthrough. Taking ncRNAs as an example, growing attention has paid to circRNAs, a newly-discovered covalently closed ncRNAs [345] who have been shown to involved in the initiation and regulation of various cancers [346–348]. Serve as a competing endogenous RNA, circRNAs have been explored to regulate splicing or transcription, bind or sequester proteins, especially regarded as the miRNA sponge [349–351]. As an illustration, circRNA NOP2/Sun RNA methyltransferase 2(circNSUN2), prominently elevated in the cytoplasm of colorectal carcinoma (CRC) cells under m6A modification, stabilizes the structure of high mobility group AT-hook 2 (HMGA2) mRNA via interacting with an RNA-binding protein called Insulin-Like Growth Factor 2 mRNA-Binding Protein 2 (IGF2BP2), ultimately enhancing the aggressive of CRC cells [352]. Meanwhile, by sponging miR-181a-5p, circNSUN2 increased the expression of Rho-associated coiled-coil-containing protein kinase 2 (ROCK2), a key molecule in cancer growth, subsequently improve the proliferation and migration of CRC cells [353] (Fig. 4). As a result, continuous exploration of such traditional epigenetic regulatory areas will not only exceedingly increase the continuous in-depth understanding of epigenetics, also optimize the development and efficacy of cancer-target drugs.

Although targeted epigenetic therapy is fruitful, epi-drugs are not broadly applicated to diverse tumors, with their unsatisfactory efficacy partly due to the low bioavailability, poor stability and relatively unbearable toxicity, as well as irregularities in dosing time and dose, owning plentiful challenges in clinic applications. For example, despite the emerging m6A-associated targets of therapeutic value have been identified in different types of infiltrated immune cells, and METTL3 has been widely verified to deeply impact tumor biology and TME harmony as the predominant catalytic subunit of m6A writer complex [117, 173, 174, 200, 201], only several small molecules targeting m6A-associated enzymes were evaluated in some preclinical trials [125, 186]. Taking together, there remaining huge blanks in the relevant area, thus developing drugs to rescue the activity or to mimic the biological function of those m6A enzymes may provide a novel insight into tumor immunotherapy.

From another perspective, all of those above aspirations should be exceedingly assisted by miscellaneous high-throughput, highly sensitive and domain-wide epigenetic research techniques, such as DNA methylation mapping of cells by genome-wide/precision DNA methylation and hydroxymethylation sequencing, capturing genome-wide interactions and high-level structures of genetic elements using various sensitive spatial genomics techniques, and improving the precision to the single-cell level to analyze the heterogeneity of epi-modifications in different cell subpopulations under complex TME, etc. Further enhancement of these research techniques and analytical methods will be of great help in elucidating TME as a key regulatory component of the tumorigenic process.

In summary, more and more evidence prove that epigenetic events play roles in tumor progression, maintenance, metastasis and prognosis, also take controls in TME remodel and regulation via complicated and dynamic mechanisms, while the potential epi-drugs along with its combination with traditional immunotherapy has rapidly grown into the prospective pattern for cancer treatment. Going forward, there are great expectations provide deeper insight into the extensive exploration and further refinement of the epigenetic processes, supplying boot opportunities for improved and newly explored therapeutic interventions in cancer.

## Data Availability

All data used to support the findings of this study are included within the article.
